# Correspondence

**Published:** 1869-11

**Authors:** 


### Correspondence.

*To the Editor of the Buffalo Medical and Surgical Journal:*

DEAR SIR,—In the last number of your valuable Journal there appears a communication from W. L. Atlee, M. D., giving my letters to him, with comments, without publishing in the same connection his letter to me. Now, Mr. Editor, I have no time, and certainly no inclination, to discuss questions of ethics or practice with Dr. Atlee, and shall very reluctantly, therefore, be drawn into any controversy on the subjects mentioned in those letters. The whole matter is very simple; and by merely publishing my letter to Dr. Atlee, and his reply, every member of the profession who may feel sufficient interest in the questions involved to read them, will be able to form his own conclusions.

Having been often importuned by a much respected patient to remove the large fibrous tumor which disfigured her, I had as often assured her that it was not of a character suitable for excision. I had told her that it would be equally proper to undertake the removal of her heart, or her lungs, if diseased, as her non-malignant uterine growth, which would, for its removal, require the excision of the greater portion of the uterus. Upon a recent visit she annoyed me by saying that some of Dr. Atlee's friends had been visiting her, that they assured her that he frequently removed such tumors, and that they insisted she should consult him.

At her request, therefore, I cheerfully wrote to Dr. Atlee the letter which follows, believing that no well informed gynecologist could hesitate in pronouncing an opinion in the premises, saying in substance, that we were not warranted in undertaking the removal of large non-malignant fibrous growths which involve the uterus to such an extent as to require for their extirpation the excision of any considerable portion of that organ.

The reply of Dr. Atlee disappointed and annoyed me. Instead of a manly and honorable avowal of the impracticability of such an operation, it seemed to me to be non-committal and evasive, leaving the impression that he wished the reader to think that he had done, and could do, as great things as that. I was charitable enough, I confess, to interpret his letter as not advising an operation in this instance, and, in my letter to it, "concurred" in that opinion. Avoiding a direct answer to my question, he volunteered the recommendation of the use of "muriate of ammonia" for the discussion of such a tumor. I thought, in so doing, he trifled with me and treated me as a neophyte.

I showed the letter to some of the most intelligent men in the country, who chanced to be at my house about that time, and they confirmed my opinion of its want of professional frankness. Among those who read the letter was yourself; and, at your request, I did not hesitate to hand it to you for publication.

I regret that I had not a copy of my letter to Dr. A., to which his was in reply, that they might have been published in connection, as I now desire. It would have obviated the necessity of my giving from memory a statement of its substance, in which I perceive there is some confusion of the statements, though unessential, of the second letter with those of the first, having made no copy of either and writing only from memory.

In relation to the private character of the letter, I submit, whether a professional opinion, given to another practitioner for a consideration, without any intimation that it should be considered private or confidential, comes within that sacred class? Or whether, if it contain important scientific improvements or principles, or is otherwise "remarkable," any rule of propriety is violated by making it public through a medical journal, or other professional media of communication? This much, Mr. Editor, was necessary in order to set forth the facts; and I am entirely willing to submit them without comment to my medical brethren and abide their verdict.

Hoping that you will again insert my hastily written letter to Dr. A., and his reply, I trust I shall not again deem it necessary to encumber your columns with a matter of so little general interest to your readers, and remain,

Truly yours, JAMES P. WHITE.

---

"Buffalo, Aug't 16, 1869.

"Dr. W. L. ATLEE:

Dear Sir, I have a friend 45 years old, widow, who has never been pregnant, in pretty good health, who has a fibrous tumor, probably intra-mural, of ten or twelve years standing, and now extending above the umbilicus. It is irregular in form, but I find no evidence in manipulating that there are adhesions, although she has suffered a good deal with superficial abdominal cramps and pains

—no hemorrhages or leucorrhea. The uterus is considerably enlarged, as shown by the sound, and crowded anteriorly.

My object in writing is, in her behalf, to ask if you would advise extirpation? If you would like any further or more minute description, I shall be happy to furnish it. Should you wish an examination, in case you advise excision, before undertaking it, she can come down and see you. Mrs. F. is highly respectable and well connected, and having a good home, would probably prefer the operation at home, in case you decide upon making it. In my opinion, the uterus is so involved in the tumor that it would require to be removed at the neck.

Some friends of yours have advised Mrs. F. that you are removing tumors of this character, and I write at her request to ask if it is true?

Should you not remember me, I will take the liberty of referring you to my friend, Dr. S. D. GROSS.

Awaiting your response, and with great respect, I remain.

Your obd't serv't,

JAMES P. WHITE."

"P. S.—Please enclose bill for fee for correspondence, and I will remit at once."

"Yours truly,

J. P. WHITE."

---

"Philadelphia, Aug. 17, 1869.

"DEAR SIR,—I rec'd yours of the 16th to-day. Certain forms of Uterine Fibroids I remove by the knife; but in deciding on the propriety of an operation in any case, it is necessary to make a careful personal examination. As the patient, however, is 'in pretty good health,' and there is 'no hemorrhage,' the case is not urgent. I would, therefore, advise medication, which sometimes will disperse these growths. Give her ten grains of muriate of ammonia three times a day; and also, twice a day, order her to wash the abdomen well with a solution of it, ℥ii to the pint of water. Persist in this treatment for a long time, provided the health remains good and the tumor does not increase. If convenient I would like to see her.

"Very respectfully yours,

"WASHINGTON L. ATLEE,

"1480 Arch St.

"To J. P. WHITE, M. D.,

"Buffalo, N. Y.

"Fee, $10."

